# Supplementary figures and images for: Protein structure and sequence re-analysis of 2019-nCoV genome does not indicate snakes as its intermediate host or the unique similarity between its spike protein insertions and HIV-1
Source: bioRxiv. 2020 Feb 8:2020.02.04.933135. Preprint. [Version 1] doi: 10.1101/2020.02.04.933135 (PMC7238725; doi:10.1101/2020.02.04.933135)

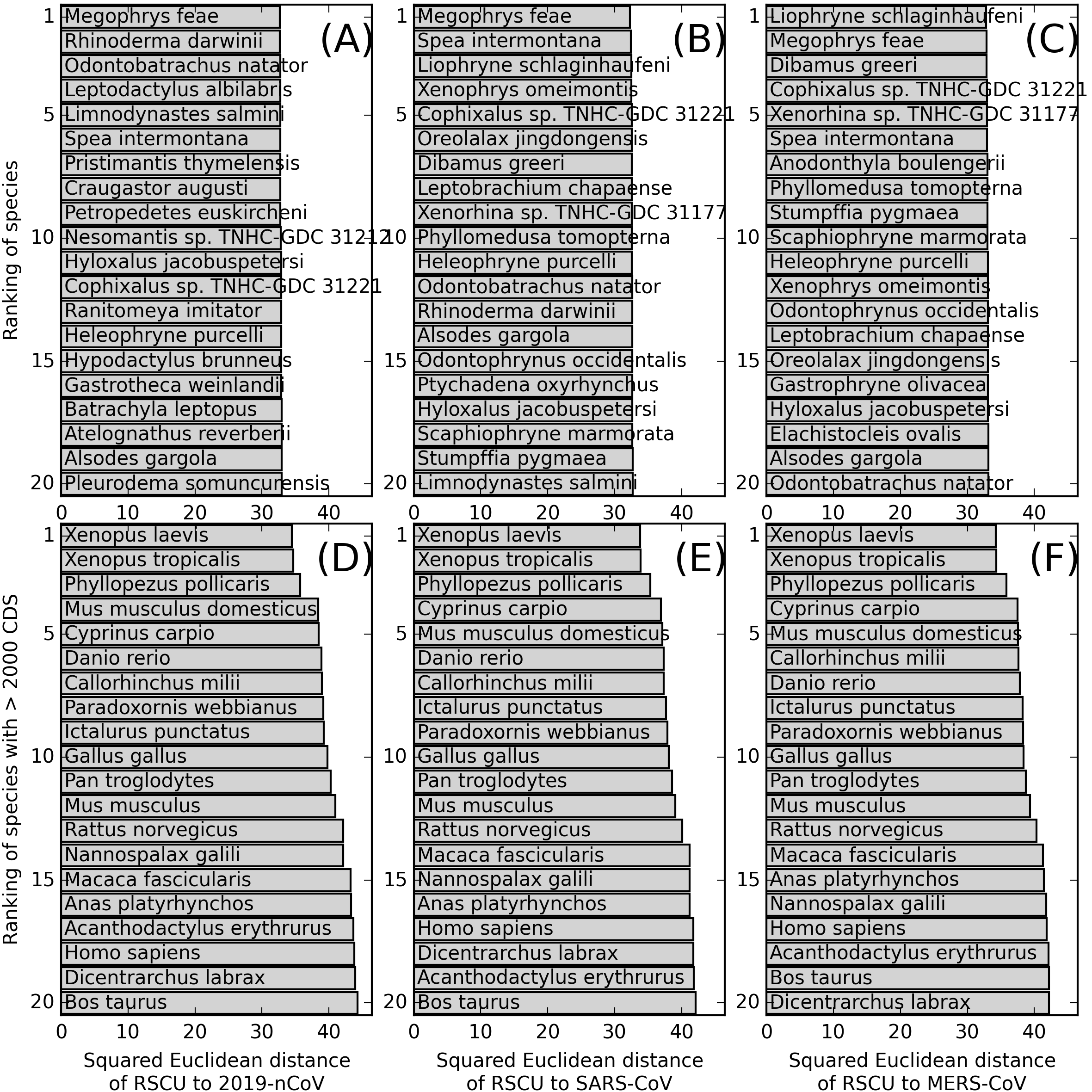

Supplement: Supplement 1 [file media-4.tif]

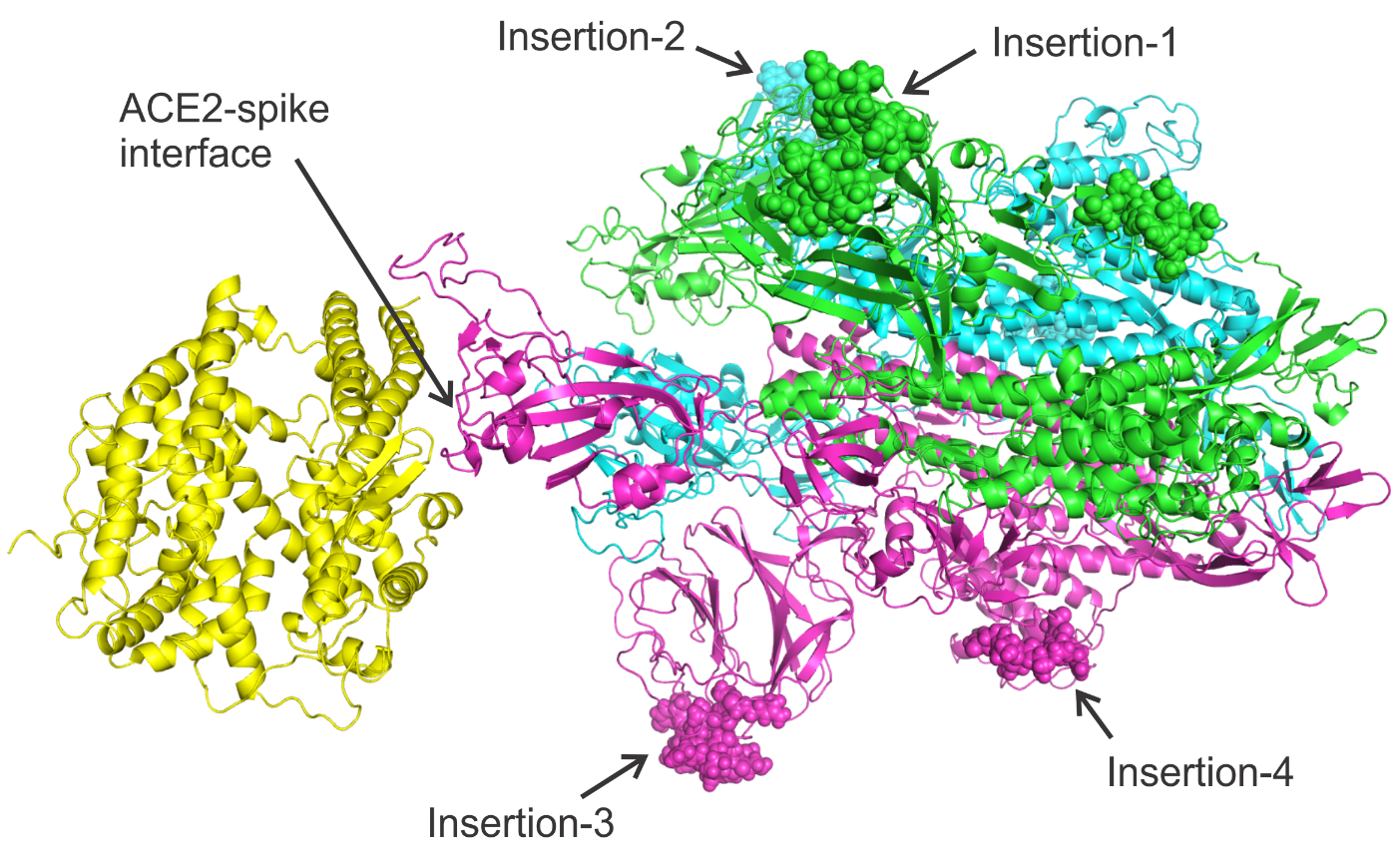

Supplement: Supplement 2 [file media-2.tif]

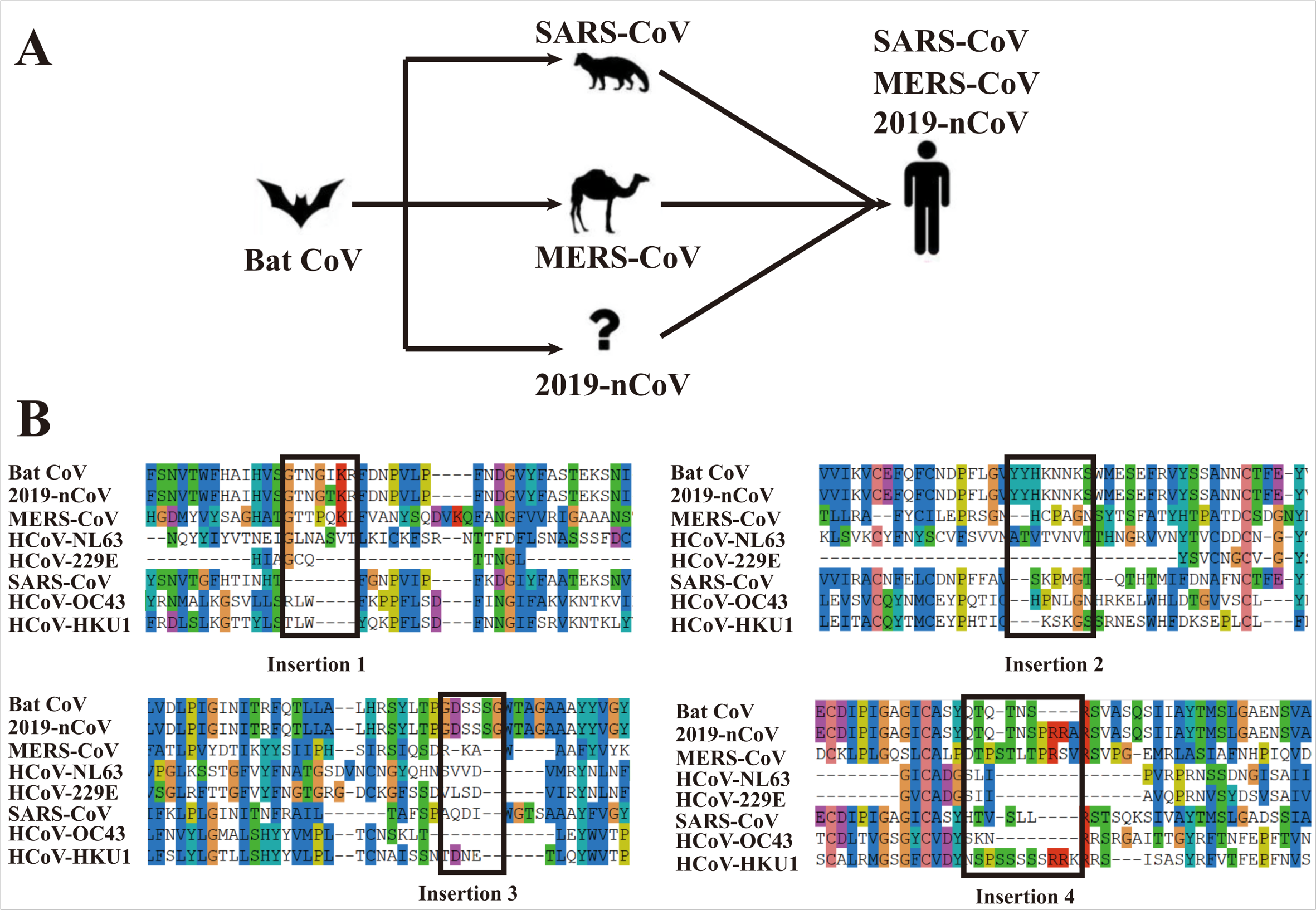

Supplement: Supplement 3 [file media-3.tif]
